# Lower Adherence to the Mediterranean Diet in Fibromyalgia Compared with Rheumatoid and Psoriatic Arthritis and Its Association with Disease Burden and Lifestyle Factors

**DOI:** 10.3390/nu18071019

**Published:** 2026-03-24

**Authors:** Cristina Iannuccelli, Martina Favretti, Giulio Dolcini, Carlo Cauli, Vincenzo Ferraro, Daniele Franculli, Giulia Scalese, Rossana Scrivo, Fabrizio Conti, Manuela Di Franco

**Affiliations:** 1 Rheumatology Unit, AOU Policlinico Umberto I, Sapienza University of Rome, 00185 Rome, Italy; iannuccelli.cristina@policlinicoumberto1.it; 2 Department of Molecular Medicine, Sapienza University of Rome, 00185 Rome, Italy; martina.favretti@uniroma1.it; 3 Rheumatology Unit, Department of Medical and Cardiovascular Sciences, Sapienza University of Rome, 00185 Rome, Italy; carlo.cauli@uniroma1.it (C.C.); vincenzo.ferraro@uniroma1.it (V.F.); rossana.scrivo@uniroma1.it (R.S.); fabrizio.conti@uniroma1.it (F.C.); manuela.difranco@uniroma1.it (M.D.F.); 4 Department of Medicine, Acquapendente Civil Hospital, 01021 Acquapendente, Italy; daniele.franculli@asl.vt.it; 5 Gastroenterology Unit, Department of Translational and Precision Medicine, Sapienza University of Rome, 00185 Rome, Italy; giulia.scalese@uniroma1.it

**Keywords:** mediterranean diet, rheumatoid arthritis, psoriatic arthritis, fibromyalgia, lifestyle factors

## Abstract

**Background**: The Mediterranean diet (MedDiet) has been associated with anti-inflammatory effects and potential benefits in several chronic conditions. However, adherence to the MedDiet and its relationship with lifestyle factors and disease severity across different rheumatological diseases remain poorly characterized. **Objectives**: This study aimed to evaluate differences in MedDiet adherence among patients with rheumatoid arthritis (RA), psoriatic arthritis (PsA), and fibromyalgia (FM), and to explore its association with cardiovascular comorbidities, bowel habits, and disease-related outcomes. **Methods**: In this monocentric cross-sectional study, adherence to the MedDiet was assessed using the 14-item PREDIMED questionnaire. Self-reported data on sociodemographic characteristics, cardiovascular comorbidities, bowel habits, and dietary behaviors were collected through questionnaires. Disease activity and severity were assessed using validated disease-specific measures. Differences in MedDiet adherence across diagnostic groups were evaluated using non-parametric tests. Multivariable models were performed to examine associations between MedDiet adherence and cardiovascular comorbidities or disease outcomes, adjusting for potential confounders including age, sex, BMI, smoking status, and educational level. **Results**: A total of 422 participants were included (RA *n* = 165, PsA *n* = 85, FM *n* = 172). Significant differences in MedDiet adherence were observed across diagnostic groups (*p* < 0.001), with the highest adherence in RA, intermediate values in PsA, and the lowest in FM. Compared with the other groups, a higher proportion of FM participants reported food intolerances (46.5%) and restrictive diets, including lactose-free (34.9%) and gluten-free (15.1%) diets. In the FM group, high adherence to the MedDiet was significantly associated with lower FIQR scores (β = −16.9; 95% CI −32.1 to −1.7; *p* = 0.01) and lower PDS scores (β = −4.34; 95% CI −7.81 to −0.86; *p* = 0.01). Sensitivity analyses using the continuous PREDIMED score confirmed these associations. **Conclusions**: Adherence to the MedDiet differs across rheumatological diseases, with the lowest adherence observed in FM. Higher adherence was associated with lower disease severity and impact in FM. These findings highlight the potential relevance of nutritional counselling in rheumatological diseases and support the need for longitudinal and interventional studies evaluating the role of the MedDiet within multidisciplinary disease management.

## 1. Introduction

Musculoskeletal diseases represent an increasing cause of disability and reduced work productivity worldwide, particularly in Europe [1]. Among these conditions, fibromyalgia (FM) [2] and inflammatory arthritis, including rheumatoid arthritis (RA) [3] and psoriatic arthritis (PsA) [4], are major contributors to chronic pain, functional impairment, and reduced quality of life.

Although effective pharmacological therapies are available, particularly for inflammatory arthritis, it is increasingly recognized that optimal disease management cannot rely solely on drug treatment. The management of FM remains particularly challenging, and patients frequently seek complementary and alternative interventions, such as dietary modifications and nutritional supplements, often in the absence of robust evidence of efficacy [5]. Within a biopsychosocial framework of chronic disease management [6], lifestyle interventions, including dietary modification, are considered essential components of comprehensive care aimed at reducing disability and limiting comorbidities. The European Alliance of Associations for Rheumatology (EULAR) has issued recommendations on lifestyle behavior in rheumatic and musculoskeletal diseases [7], encouraging adherence to a healthy, balanced diet in line with World Health Organization guidelines. However, no specific dietary recommendations were proposed due to limited evidence.

The Mediterranean diet (MedDiet), a traditional dietary pattern common in Mediterranean countries [8], is characterized by high consumption of vegetables, fruits, nuts, legumes, and minimally processed grains; moderate consumption of fish and shellfish; and low or very low consumption of meat, particularly red meat, and whole milk products, with the exception of fermented dairy products such as cheese and yogurt. Extra virgin olive oil is the primary source of dietary fat, while moderate red wine consumption during meals represents the main source of alcohol [9]. This dietary pattern has been extensively associated with reduced cardiovascular risk and lower all-cause mortality [10], likely through the synergistic effects of its anti-inflammatory and antioxidant components rather than any single nutrient.

Several components of the MedDiet may exert beneficial effects in rheumatic diseases by modulating inflammatory and metabolic pathways [11]. Polyphenols present in olive oil have anti-inflammatory and antioxidant effects; omega-3 polyunsaturated fatty acids derived from fatty fish can modulate eicosanoid pathways and immune responses [12]; and dietary fiber promotes gut microbiota diversity and short-chain fatty acid production, which support regulatory immune mechanisms [13]. These mechanisms provide a biologically plausible link between adherence to the MedDiet and modulation of systemic inflammation and chronic pain. Furthermore, the MedDiet may be particularly relevant for patients with rheumatic diseases given their increased burden of cardiometabolic comorbidities [14].

However, clinical evidence remains heterogeneous. In RA, some studies have reported improvements in pain [15] and disease activity [16,17] following MedDiet interventions, although effects on objective inflammatory markers such as C-reactive protein (CRP) have been inconsistent [18]. In PsA, data regarding the association between MedDiet adherence and disease activity remains inconsistent [19,20], and interventional data remains scarce and limited by small sample sizes and short follow-up [21]. In FM, preliminary evidence indicates potential benefits on symptom severity and pain intensity [22], possibly mediated by improvements in oxidative stress and gut dysbiosis, although robust controlled trials are lacking [23].

Notably, a substantial proportion of patients with immune-mediated inflammatory diseases [24,25] and FM [26] report modifying their diet habits after diagnosis, often without structured nutritional guidance. Nevertheless, these changes frequently involve self-initiated restrictive diets [25], and studies evaluating adherence to the MedDiet in RA [17,27], PsA [19,20], and FM [28] have consistently reported low-to-moderate adherence levels, even in Mediterranean countries.

Despite the growing interest in nutritional approaches in rheumatology, comparative data on dietary patterns across different rheumatic diseases remain scarce [29]. To date, no study has systematically compared adherence to MedDiet across inflammatory arthritis and FM within the same clinical setting. Furthermore, the relationship between dietary adherence and factors such as bowel habits and cardiovascular comorbidities remains poorly characterized.

Therefore, the primary aim of this study was to compare adherence to MedDiet among patients with RA, PsA, and FM. Secondary aims were to explore potential associations between dietary adherence and cardiovascular risk factors, bowel habits, and disease activity or symptom severity within each diagnostic group.

## 2. Materials and Methods

### 2.1. Study Design and Setting

This observational cross-sectional study was conducted on a monocentric cohort of patients attending the Rheumatology Unit of the AOU Policlinico Umberto I, Rome, between January and December 2025.

Patients with RA and PsA on stable therapy prescribed according to international recommendations were consecutively recruited during routine outpatient visits. Patients with FM were identified from the same clinical setting based on visits performed within the previous month.

### 2.2. Participants

Adult patients (≥18 years) with an established diagnosis of RA, according to the 2010 American College of Rheumatology (ACR)/EULAR classification criteria [30], or PsA, according to the Classification Criteria for Psoriatic Arthritis (CASPAR) [31], were eligible for inclusion. These patients completed paper-based questionnaires during routine outpatient visits without assistance from healthcare personnel.

Patients with FM were included if they were aged ≥18 years and had a confirmed diagnosis of FM according to the 2016 ACR criteria [32]. In this group, questionnaires were administered online and included instruments assessing disease severity and impact.

For all groups, only patients receiving stable pharmacological and non-pharmacological treatment for at least one month prior to data collection were included.

Exclusion criteria were active pregnancy, acute intercurrent diseases, inability to complete the questionnaires, and concomitant diagnosis of inflammatory arthritis and FM.

### 2.3. Data Collection

Data were collected through a structured questionnaire, administered in paper format during outpatient visits for AR and PsA patients; for FM patients, questionnaires were administered in online format, within one month of the last visit.

Clinical and serological data, including disease activity measures, were retrieved from medical records. For patients with RA and PsA, disease activity corresponded to the clinical assessment performed during the same outpatient visit in which the questionnaire was completed. For patients with FM, data not collected online were retrieved from the most recent clinical evaluation performed within the previous month.

### 2.4. Questionnaires and Variables

#### 2.4.1. Lifestyle Variables

The first section of the questionnaire collected sociodemographic and self-reported clinical information, including age, sex, education level, occupational status, living area (rural vs. urban), and smoking status. Information on alcohol consumption was also collected as a categorical variable (current consumption vs. no consumption). However, quantitative data regarding the amount or type of alcoholic beverages consumed were not recorded.

Additional information on bowel habits, food intolerances, use of local food products, and adherence to lactose-free or gluten-free diets was also collected.

Self-reported comorbidities included coeliac disease, psoriasis, hypertension, dyslipidemia, and type II diabetes mellitus.

Anthropometric data (height and weight) were self-reported and used to calculate body mass index (BMI), defined as weight (kg) divided by height squared (m^2^). BMI was categorized as normal weight (<25 kg/m^2^), overweight (25–29.9 kg/m^2^), or obese (≥30 kg/m^2^).

All sociodemographic and clinical information regarding concomitant conditions was self-reported, and no objective measurements of blood pressure, lipid profile, or glycemic control were required for study participation.

#### 2.4.2. Adherence to the Mediterranean Diet

The second section of the questionnaire assessed adherence to the MedDiet using the 14-item PREvención con DIeta MEDiterránea (PREDIMED) questionnaire.

The PREDIMED questionnaire is a tool originally developed in a Spanish study on myocardial infarction [33]. The original instrument was composed of nine items evaluating key components of the Mediterranean dietary pattern, including olive oil use and consumption of fruits, vegetables, legumes, fish, nuts, meats, whole grains, and red wine.

Five additional items were subsequently included [34]. Two of these items evaluate dietary habits, particularly the use of olive oil as the main culinary fat and the preference of white meat over red meat. The remaining items assess the frequency of consumption of nuts, sugar-sweetened beverages, and a traditional Mediterranean tomato-based sauce (soffritto). Each item is scored as 0 or 1, yielding a score ranging from 0 to 14, with higher scores indicating greater adherence to the MedDiet. Based on predefined cut-offs, adherence can be categorized as low (0–5 points), moderate (6–9 points), and high (10–14 points) [35].

#### 2.4.3. Disease Activity

Disease activity was assessed using validated, disease-specific instruments.

In patients with RA, disease activity was evaluated using the CRP-based Disease Activity Score 28 (DAS28), a composite index derived from the twenty-eight tender joint count (TJC), twenty-eight swollen joint count (SJC), patient global assessment (PtGA), and CRP [36].

In patients with PsA, disease activity was assessed using the Disease Activity index for Psoriatic Arthritis (DAPSA), calculated as the sum of TJC68, SJC66, PtGA, patient pain assessed on a 10-cm visual analogue scale (VAS), and CRP [37].

In patients with FM, disease severity and disease impact were assessed using the Polysymptomatic Distress Scale (PDS) and the Revised Fibromyalgia Impact Questionnaire (FIQR), respectively. The PDS is calculated as the sum of the Widespread Pain Index (WPI) and the Symptom Severity Scale (SSS) [38]. The FIQR is a multidimensional questionnaire composed of three subdomains evaluating physical function, overall disease impact, and symptom severity [39].

### 2.5. Ethical Considerations

The study protocol was approved by the Ethics Committee of Sapienza University (Lazio Area 1 Ethics Committee) as an amendment to study no. 7658 (prot. 0087/2026).

The study was conducted in accordance with the principles of the Declaration of Helsinki, and all patients provided written informed consent prior to participation.

### 2.6. Statistical Methods

#### 2.6.1. Sample Size Calculation

A minimum sample size calculation was performed to ensure that the study was adequately powered to detect differences in MedDiet adherence across the three diagnostic groups. The primary outcome of the study was the comparison of MedDiet adherence between groups, evaluated using both the continuous PREDIMED score and its predefined adherence categories.

In the absence of reliable prior estimates, a medium effect size (Cohen’s *f* = 0.25), was assumed. Considering a significance level of α = 0.05, a statistical power of 85%, and three comparison groups, the estimated minimum required sample size was 180 participants (60 per group) based on a one-way ANOVA model.

For categorical comparisons based on a chi-squared test assuming three diagnostic groups and three categories of dietary adherence, the estimated minimum sample size was 216 participants (72 per group).

The final study population included 422 participants, therefore exceeding the minimum required sample size.

The sample size calculation was performed using G*Power software (version 3.1.9.7, Heinrich-Heine-University Düsseldorf, Germany).

#### 2.6.2. Statistical Analysis

Continuous variables are reported as median and interquartile range (IQR), while categorical variables are expressed as absolute and relative frequencies.

The distribution of sociodemographic characteristics, cardiovascular comorbidities, and dietary habits across diagnostic groups was explored using univariate analyses. Continuous variables, which were not normally distributed, were compared across the three diagnostic groups using the Kruskal–Wallis test, followed by Dunn’s post hoc test with Bonferroni correction. Categorical variables were compared using either Fisher’s exact test or the chi-squared test, depending on expected cell counts, with Bonferroni adjustment for multiple comparisons.

For exploratory association analyses, adherence to the MedDiet was primarily analyzed as a categorical variable based on the predefined PREDIMED score thresholds. This categorization was used to facilitate interpretation of adherence levels and comparison with previous literature. To assess the robustness of the findings, additional analyses were also performed using the PREDIMED score as a continuous variable.

To evaluate associations between cardiovascular risk factors and categories of dietary adherence, multivariable logistic regression models were performed. Cardiovascular risk factors (smoking habit, BMI > 25 kg/m^2^, hypertension, dyslipidemia) and impaired bowel function were considered as dependent variables, while dietary adherence category was the main independent variable. Models were adjusted for potential confounders identified a priori based on clinical relevance, including age, sex, and education level. Analyses were conducted both in the overall population, including diagnosis as a covariate, and stratified by disease group.

Associations between dietary adherence and disease activity or severity measures were assessed using multivariable linear regression models. Disease activity or severity measures were considered as dependent variables (DAS28-CRP for RA, DAPSA for PsA, and FIQR and PDS for FM, as well as PtGA and VAS pain scores for RA and PsA). The main independent variable was dietary adherence category. These models were adjusted for age, sex, education level, smoking habit, and BMI.

Missing data were handled using multiple imputations by chained equations (MICE), generating five imputed datasets for each outcome. Regression coefficients and standard errors were pooled across imputations according to Rubin’s rules.

Effect estimates are reported as regression coefficients or odds ratios (ORs) with 95% confidence intervals (CIs). A two-sided *p*-value < 0.05 was considered statistically significant.

All statistical analyses were conducted using R software (version 4.4.3).

## 3. Results

### 3.1. Participant Characteristics

A total of 422 participants were included in the study: 165 with RA, 85 with PsA, and 172 with FM. Baseline characteristics are summarized in Table 1 and Table 2.

Patients with FM were younger and more frequently female compared with those with RA and PsA, and they also showed a higher education level. Regarding disease activity, patients with RA and PsA were in remission or low disease activity (median DAS28-CRP 2.36 [IQR 1.55]; median DAPSA 7.14 [IQR 11.72]), whereas FM patients showed high disease severity according to the FIQR score (median 65.0 [IQR 26.95]).

Cardiovascular comorbidities were broadly comparable across groups, although PsA patients showed a slightly higher BMI compared with RA and FM patients (Table 2).

### 3.2. Mediterranean Diet Adherence Across Diagnostic Groups

**Figure 1:**
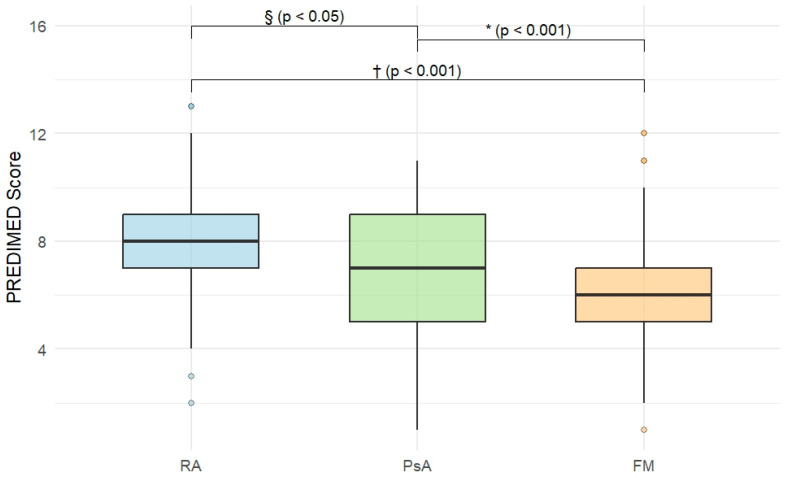
Box-plot showing PREDIMED scores differences among the three diagnostic groups.

**Figure 2:**
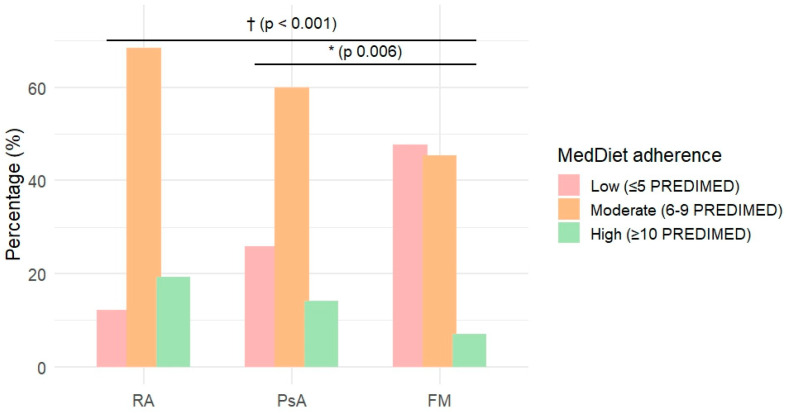
Distribution of the PREDIMED adherence category among the three diagnostic groups.

MedDiet adherence differed significantly across diagnostic groups, showing a progressive decrease from RA to PsA and FM (Table 3; Figure 1 and Figure 2).

FM patients exhibited lower PREDIMED scores compared with both RA and PsA patients (median 6.0 [IQR 2.0] vs. 8.0 [2.0] and 7.0 [4.0], respectively; *p* < 0.001). Consistently, the distribution of adherence categories differed significantly across groups (*p* < 0.001), with nearly half of FM patients classified in the low-adherence category (47.7%), compared with 12.1% of RA and 25.9% of PsA patients.

Univariate analyses also showed that food intolerances and restrictive dietary patterns were more frequently reported by FM patients compared with the other groups (Table 3).

### 3.3. Associations Between Mediterranean Diet Adherence and Cardiovascular Risk Factors or Bowel Habits

Multivariable logistic regression analyses were conducted to explore associations between MedDiet adherence and cardiovascular risk factors (Supplementary Table S1). No significant associations were observed between adherence categories and smoking habit, overweight/obesity, hypertension, or dyslipidemia.

However, moderate adherence to the MedDiet was associated with a lower likelihood of bowel habit alterations in the overall population (OR 0.46; 95% CI 0.28–0.76; *p* = 0.002). A similar association was observed in the RA subgroup (OR 0.26; 95% CI 0.09–0.72; *p* = 0.01). These associations were not confirmed when the PREDIMED score was analyzed as a continuous variable (Supplementary Table S2).

### 3.4. Associations Between Mediterranean Diet Adherence and Disease Activity

**Figure 3:**
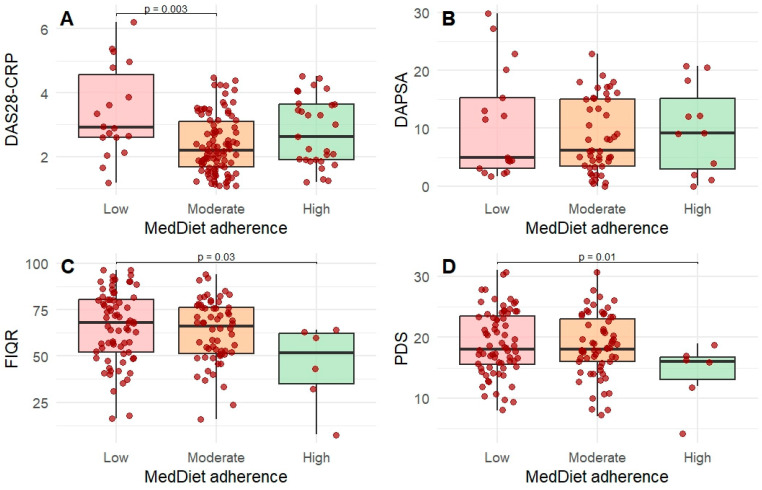
Box-plot showing disease activity and severity scores differences between dietary adherence category among the three diagnostic groups. Points represent observed (non-imputed) data used for visualization, while regression analyses were performed on multiply imputed datasets. (**A**) disease activity differences between dietary adherence category in AR group (**B**) disease activity differences between dietary adherence category in PsA group (**C**,**D**) disease impact and disease severity differences between dietary adherence category in FM group.

Multivariable linear regression models adjusted for age, sex, BMI, smoking habit, and education level were used to evaluate associations between MedDiet adherence and disease activity or symptom severity. Regression analyses were performed using multiply imputed datasets (m = 5), corresponding to the full study population (Table 4; Figure 3).

In patients with RA (*n* = 165), moderate adherence to the MedDiet was associated with lower DAS28-CRP compared with low adherence (β = −0.90; 95% CI −1.50 to −0.31; *p* = 0.003). Similarly, moderate adherence was associated with lower PtGA (β = −1.57; 95% CI −3.00 to −0.15; *p* = 0.03). However, these associations were not confirmed when the PREDIMED score was analyzed as a continuous variable (Supplementary Table S2).

In patients with FM (*n* = 172), high adherence to the MedDiet was associated with lower FIQR scores compared with low adherence (β = −16.9; 95% CI −32.1 to −1.7; *p* = 0.01) and lower PDS scores (β = −4.34; 95% CI −7.81 to −0.86; *p* = 0.01). Sensitivity analyses using the continuous PREDIMED score confirmed these associations (FIQR β = −1.96; 95% CI −3.62 to −0.30; *p* = 0.02; PDS β = −0.64; 95% CI −1.12 to −0.16; *p* = 0.009) (Supplementary Table S3).

Although the number of FM patients with high adherence was small (*n* = 12), the consistency of the results in analyses using the continuous PREDIMED score supports the robustness of these findings.

## 4. Discussion

This monocentric observational cross-sectional study evaluated adherence to the MedDiet among patients with RA, PsA, and FM, identifying marked differences across these diagnostic groups. A clear gradient emerged, with the highest adherence observed in RA, intermediate levels in PsA, and the lowest adherence in FM. Several factors may contribute to these differences.

A growing body of literature has explored the relationship between dietary habits and inflammatory arthritis [40], particularly RA [41]. This increasing scientific attention may contribute to greater awareness among both clinicians and patients regarding the potential role of diet in disease management. Previous studies suggest that patients with inflammatory arthritis often consider nutrition a key component of disease management and frequently modify their dietary habits after diagnosis [24,25]. In a study evaluating nutritional knowledge in patients with inflammatory arthritis, nearly 60% of participants reported awareness of the potential role of diet in disease control and management of comorbidities [42]. Nevertheless, relevant knowledge gaps remain, particularly regarding specific foods to consume or avoid. Importantly, although many patients report changing their diet autonomously, a substantial proportion also report receiving some form of dietary advice from physicians [24]. These factors may partly explain the relatively higher adherence to the MedDiet observed in the RA group in our cohort.

In contrast, the relationship between nutrition and FM remains less clearly defined [43], and patients with FM often rely on non-medical sources for dietary information, including television [44] and the internet [45]. In our cohort, a considerable proportion of FM patients reported food intolerances (46.5%) and reported following restrictive diets such as lactose-free (34.9%) or gluten-free (15.1%), despite only a small proportion having a confirmed diagnosis of coeliac disease. In our study, we did not systematically record whether diets were prescribed by healthcare professionals or self-initiated. However, previous research suggests that restrictive diets in FM are frequently self-prescribed or influenced by nonmedical sources [46]. These dietary patterns may represent a potential confounding factor contributing to lower overall adherence to the MedDiet observed in this group.

It should also be considered that, in our study, questionnaires were administered in-clinic for RA and PsA patients and online for FM patients. Although in-clinic questionnaires were self-administered without assistance from healthcare personnel, which likely reduced interviewer-related bias, the clinical setting itself may still have influenced responses through social desirability. In contrast, online administration may have allowed for greater anonymity and more candid reporting. These differences in administration modality could have introduced systematic measurement and response bias, potentially contributing to the lower PREDIMED scores observed in the FM group and amplifying between-group differences. Furthermore, the lack of supervision in the online setting may have increased the risk of misunderstanding or less accurate responses. Therefore, the observed differences should be interpreted with caution, as they may partly reflect methodological differences.

The high prevalence of a lactose-free diet observed in our study is consistent with previous literature reporting a higher frequency of lactose intolerance in FM patients, although prevalence estimates vary substantially depending on whether intolerance is self-reported or objectively diagnosed. Prevalence rates as high as 36.5% have been reported when based on self-report [47], compared with approximately 4% when confirmed through diagnostic testing [26]. In our cohort, the specific type of food intolerance was not recorded, preventing a more detailed analysis. This represents an important limitation, as some of these dietary restrictions may influence the ability to adhere to traditional Mediterranean dietary patterns.

The adoption of gluten-free diets in the absence of confirmed coeliac disease may also reflect attempts to improve FM symptoms. Some small studies have suggested potential improvements in pain or functional symptoms following gluten-free diets, particularly in patients with gluten sensitivity [48,49]. However, these studies were limited by small sample sizes and the absence of a placebo control. A larger controlled trial has produced less consistent results, with only a minority of patients showing clinical improvement [50]. Overall, current evidence remains insufficient to support routine use of gluten-free diets in FM, and further randomized controlled trials are needed to clarify whether specific patients’ subgroups may benefit from targeted dietary interventions [51].

Within this context, adherence to a balanced dietary pattern such as the MedDiet may represent a potentially relevant component of lifestyle in FM. In our study, higher adherence to the MedDiet was associated with lower disease severity and impact among FM patients. However, these findings should be interpreted with caution, considering the cross-sectional design of the study but also potential residual confounding. In particular, unmeasured factors such as physical activity, psychological status, health awareness, and overall lifestyle behaviours may be associated with both dietary habits and symptom severity. Therefore, the observed associations may reflect a broader healthy lifestyle profile rather than a direct effect of diet alone. Our results are consistent with previous cross-sectional studies reporting associations between higher MedDiet adherence and lower disease impact in FM [29].

Several mechanisms may contribute to this association. One possible pathway involves gut microbiota. Increasing evidence suggests that gut dysbiosis may play a role in the pathophysiology of FM [52] by promoting immune activation and altered metabolite profiles that could contribute to pain hypersensitivity [53]. In this context, dietary patterns rich in fiber, antioxidants, and anti-inflammatory components, such as the MedDiet, may potentially influence gut microbiota composition. However, gut microbiome data were not available in our study, and these mechanisms remain speculative. A second possible explanation involves psychological and lifestyle factors. A previous study has reported associations between higher consumption of fruits, vegetables, and fish and better psychological outcomes in FM patients [54]. It is therefore possible that adherence to the MedDiet may be part of a broader healthy lifestyle pattern associated with better psychological well-being. Moreover, individuals with healthier dietary habits may also engage more frequently in other beneficial behaviours, such as regular physical activity [55], which represents the only strongly recommended non-pharmacological intervention for FM according to EULAR recommendations [56]. As psychological variables and physical activity levels were not assessed in our study, their potential mediating role could not be explored, and residual confounding cannot be excluded.

Regarding inflammatory arthritis, the association observed in RA when using categorical MedDiet adherence was not confirmed when the PREDIMED score was analyzed as a continuous variable. Previous cross-sectional studies have reported associations between higher adherence to the MedDiet and lower disease activity [57] or improved patient-reported outcomes [58]. However, these studies often did not fully account for lifestyle-related confounding factors. The discrepancy observed in our analyses may reflect both statistical variability and the distributional characteristics of the PREDIMED score in our sample. Most patients, particularly in the RA subgroup, were concentrated within the moderate adherence category, resulting in restricted variability and reduced sensitivity of continuous models to detect linear trends. Under these conditions, categorical analyses may better capture potential threshold effects, suggesting that the relationship between diet quality and outcomes may not be strictly linear. Nevertheless, these findings should be considered exploratory and require confirmation in larger studies.

In contrast, no significant associations were observed between MedDiet adherence and disease outcomes in PsA. These findings are consistent with previous observational [20] and interventional [21] studies. One possible explanation is that improvements in disease activity in PsA may be more strongly related to weight loss rather than to specific dietary patterns per se [59]. Alternatively, the absence of significant associations in our study may also be related to the relatively smaller sample size of the PsA group.

Several limitations should be acknowledged. First, the cross-sectional design precludes any causal inference. Second, most variables were self-reported, introducing potential recall and reporting bias. Third, differences in data collection methods across diagnostic groups may have introduced measurement and response bias. Fourth, although multivariable models adjusted for several confounders, residual confounding cannot be excluded, particularly from unmeasured factors such as physical activity, psychological status, and overall lifestyle behaviours. These aspects are especially relevant in FM, where symptom severity is strongly influenced by psychosocial and behavioural determinants, and may contribute to the observed associations. In addition, detailed information on specific food intolerances and quantitative dietary intake was not collected, limiting the ability to explore how restrictive diets may influence adherence to the MedDiet. Finally, the monocentric design and the limited number of patients in some categories may affect the generalizability of the findings.

## 5. Conclusions

In conclusion, our study identified significant differences in adherence to the MedDiet across three rheumatologic conditions, with higher adherence observed in RA and progressively lower levels in PsA and FM. In FM patients, higher adherence to the MedDiet was associated with lower disease severity and impact, although the cross-sectional design precludes causal interpretation. These findings highlight the potential relevance of nutritional factors in rheumatologic diseases and support the importance of considering dietary counseling as part of a comprehensive and multidisciplinary management approach. Future longitudinal and interventional studies are needed to clarify whether improving adherence to MedDiet may contribute to clinically meaningful improvements in disease activity, symptom burden, and quality of life. In particular, randomized controlled trials investigating structured nutritional interventions and considering potential mediating factors such as physical activity, psychological status, and gut microbiota are warranted.

## Figures and Tables

**Table 1:** Distribution of the sociodemographic and clinical characteristics among the three diagnostic groups.

	RA (*n* = 165)	PsA (*n* = 85)	FM (*n* = 172)	*p*-Value
Age (median; IQR)	62.0; 16.0 ^†^	60.0; 13.0 *	53.0; 16.0	<0.001
Female sex (*n*; %)	130; 78.8 ^†^	61; 71.8 *	158; 91.9	<0.001
Educational level (*n*; %)				
Elementary/Middle school	31; 19.6	15; 18.1 *	25; 14.5	
High school	80; 50.6	48; 57.8 *	71; 41.3	
Degree/Post-grad	47; 29.7	20; 24.1 *	76; 44.2	0.01
Occupational status (*n*; %)				
Employed	63; 38.7 ^†^	55; 65.5 *^§^	116; 67.8	
Unemployed	9; 5.5 ^†^	1; 1.2 *^§^	19; 11.1	
Retired	70; 42.9 ^†^	18; 21.4 *^§^	21; 12.3	
Housewife	21; 12.9 ^†^	10; 11.9 *^§^	15; 8.8	<0.001
Residential area (*n*; %)				
Urban area	137; 84.0	71; 84.5	146; 84.9	
Rural area	26; 16.0	13; 15.5	26; 15.1	ns
Disease activity (median; IQR)				
DAS28-CRP	2.36; 1.55	-	-	
DAPSA	-	7.14; 11.72	-	
PDS	-	-	18.00; 7.5	
FIQR	-	-	65.00; 26.95	
Patient assessment (median; IQR)				
PtGA (0–100 mm)	4.0; 4.0	3.0; 4.0	-	ns
VAS pain (0–10 cm)	4.0; 5.0	3.0; 5.0	-	ns

^†^ RA vs. FM < 0.05 (post hoc test adjusted for Bonferroni correction). * PsA vs. FM < 0.05 (post hoc test adjusted for Bonferroni correction). ^§^ PsA vs. RA < 0.05 (post hoc test adjusted for Bonferroni correction). Abbreviations: Disease Activity Score28-C Reactive Protein (DAS28-CRP), Disease Activity Index for Psoriatic Arthritis (DAPSA), Fibromyalgia (FM), Revised Fibromyalgia Impact Questionnaire (FIQR), Interquantile Range (IQR), Patient Global Assessment (PtGA), Polysymptomatic Distress Scale (PDS), Psoriatic Arthritis (PsA), Rheumatoid Arthritis (RA), Visual Analogical Scale (VAS).

**Table 2:** Distribution of cardiovascular risk factors in three diagnostic groups.

	RA (*n* = 165)	PsA (*n* = 85)	FM (*n* = 172)	*p*-Value
Smoking habit (*n*; %)	35; 21.3	22; 26.5	40; 23.4	ns
Alcohol consumption (*n*; %)	98; 60.1	51; 61.4	99; 57.6	ns
BMI (median; IQR)	24.20; 5.10	25.30; 6.10 *^§^	24.20; 6.24	0.01
BMI (*n*; %)				0.03
Normal weight	94; 59.9	34; 42.0 ^§^	97; 56.7	
Overweight	47; 29.9	28; 34.6 ^§^	52; 30.4	
Obese	16; 10.2	19; 23.5 ^§^	22; 12.9	
Hypertension (*n*; %)	47; 28.5	30; 35.3	41; 23.8	ns
Dyslipidemia (*n*; %)	32; 19.4	13; 15.3	41; 23.8	ns
Diabetes mellitus II (*n*; %)	13; 8.3	8; 9.8	14; 8.9	ns
Cutaneous psoriasis (*n*; %)	11; 7.0	54; 63.5 *^§^	15; 8.7	<0.001

* PsA vs. FM < 0.05 (post hoc test adjusted for Bonferroni correction). ^§^ PsA vs. RA < 0.05 (post hoc test adjusted for Bonferroni correction). Abbreviations: Body Mass Index (BMI), Fibromyalgia (FM), Interquantile Range (IQR), Psoriatic Arthritis (PsA), Rheumatoid Arthritis (RA).

**Table 3:** Distribution of dietary habits among the three diagnostic groups.

	RA (*n* = 165)	PsA (*n* = 85)	FM (*n* = 172)	*p*-Value
MedDiet adherence (*n*; %)				<0.001
Low (≤5 PREDIMED)	20; 12.1 ^†^	22; 25.9 *	82; 47.7	
Moderate (6–9 PREDIMED)	113; 68.5 ^†^	51; 60.0 *	78; 45.3	
High (≥10 PREDIMED)	32; 19.4 ^†^	12; 14.1 *	12; 7.0	
PREDIMED score (median; IQR)	8.0; 2.00 ^†^	7.0; 4.0 *^§^	6.0; 2.0	<0.001
Bowel habit (*n*; %)				<0.001
Regular	105; 64.8 ^†^	57; 67.1 *	60; 35.5	
Constipation (<1 time every 3 days)	16; 9.9 ^†^	10; 11.8 *	39; 23.1	
Alternate/Diarrhea (>3 times every day)	41; 25.3 ^†^	18; 21.2 *	70; 41.4	
Celiac disease (*n*; %)	4; 2.5	2; 2.4	6; 3.5	ns
Food intolerance (*n*; %)	24; 14.5 ^†^	9; 10.6 *	80; 46.5	<0.001
Specific diet (*n*; %)				
Gluten free	4; 2.5 ^†^	6; 7.1	26; 15.1	<0.001
Lactose free	23; 14.2 ^†^	15; 17.6 *	60; 34.9	<0.001
Local products (*n*; %)				
Access	134; 83.8	68; 84.0	142; 82.6	ns
Frequent use (≥3 times every week)	104; 81.9	53; 80.3	111; 78.7	ns

^†^ RA vs. FM < 0.05 (post hoc test adjusted for Bonferroni correction). * PsA vs. FM < 0.05 (post hoc test adjusted for Bonferroni correction) § PsA vs. RA < 0.05 (post hoc test adjusted for Bonferroni correction) Abbreviations: Fibromyalgia (FM), Interquantile Range (IQR), PREvención con DIeta MEDiterránea (PREDIMED), Psoriatic Arthritis (PsA), Rheumatoid Arthritis (RA).

**Table 4:** Association between MedDiet adherence categories and disease activity or severity between each diagnostic group.

	Low vs. Moderate Adherence		Low vs. High Adherence	
	β (CI 95%)	*p*-Value *	β (CI 95%)	*p*-Value *
**RA**				
DAS28-CRP	−0.90 (−1.5; −0.31)	0.003	−0.62 (−1.25; 0.01)	0.05
PtGA	−1.57 (−3.0; −0.15)	0.03	−0.93 (−2.53; 0.65)	0.24
VAS pain	−1.32 (−2.93; 0.28)	0.10	−0.76 (−2.54; 1.01)	0.39
**PsA**				
DAPSA	−1.84 (−6.17; 2.48)	0.39	−1.20 (−7.03; 4.61)	0.67
PtGA	−0.78 (−2.47; 0.90)	0.35	−0.30 (−2.52; 1.91)	0.78
VAS pain	−0.37 (−2.19; 1.43)	0.67	0.65 (−1.75; 3.06)	0.58
**FM**				
FIQR	−1.61 (−7.73; 4.51)	0.60	−16.90 (−32.1; −1.7)	0.03
PDS	−0.60 (−2.46; 1.25)	0.52	−4.34 (−7.81; −0.86)	0.01

* Model adjusted for age, sex, BMI, smoking habit, and educational level. Abbreviations: Disease Activity Score28-C Reactive Protein (DAS28-CRP), Disease Activity Index for Psoriatic Arthritis (DAPSA), Fibromyalgia (FM), Revised Fibromyalgia Impact Questionnaire (FIQR), Patient Global Assessment (PtGA), Polysymptomatic Distress Scale (PDS), Psoriatic Arthritis (PsA), Rheumatoid Arthritis (RA), Visual Analogical Scale (VAS).

## Data Availability

The original contributions presented in this study are included in the article. Further inquiries can be directed to the corresponding author.

## Supplementary Materials

The following supporting information can be downloaded at: https://www.mdpi.com/article/10.3390/nu18071019/s1.
