# Supplementary material for: Lower Adherence to the Mediterranean Diet in Fibromyalgia Compared with Rheumatoid and Psoriatic Arthritis and Its Association with Disease Burden and Lifestyle Factors
Source: Nutrients. 2026 Mar 24;18(7):1019. doi: 10.3390/nu18071019 (PMC13074341; doi:10.3390/nu18071019)
Supplement: Supplementary file 1 [file nutrients-18-01019-s001.zip › nutrients-4168510-supplementary.pdf]

Supplementary table S1. Association between MedDiet adherence categories and cardiovascular risk factors/bowel habits

	Smoking habit		BMI > 25 kg/m^2		Hypertension		Dyslipidemia		Irregular bowel habit	
Total population	OR (CI 95%)	*p-value**	OR (CI 95%)	*p-value**	OR (CI 95%)	*p-value**	OR (CI 95%)	*p-value**	OR (CI 95%)	*p-value**
Low vs Moderate Adherence	1.07 (0.6-1.8)	0.79	1.42 (0.8-2.3)	0.16	1.55 (0.8-2.7)	0.12	1.36 (0.7-2.5)	0.31	**0.46 (0.2-0.7)**	**0.002**
Low vs High Adherence	0.42 (0.1-1.0)	0.08	0.98 (0.4-2.0)	0.97	0.70 (0.2-1.6)	0.44	0.91 (0.3-2.2)	0.84	0.71 (0.3-1.4)	0.36
RA										
Low vs Moderate Adherence	1.25 (0.4-4.8)	0.71	2.04 (0.6-7.0)	0.22	2.24 (0.6-9.0)	0.20	2.31 (0.6-11.5)	0.24	**0.26 (0.0-0.7)**	**0.01**
Low vs High Adherence	0.42 (0.0-2.2)	0.31	1.62 (0.4-6.4)	0.46	1.18 (0.2-5.5)	0.82	1.29 (0.2-7.5)	0.75	0.31 (0.0-1.0)	0.06
PsA										
Low vs Moderate Adherence	0.41 (0.1-1.2)	0.12	2.03 (0.5-7.5)	0.26	2.87 (0.7-12.4)	0.12	3.94 (0.7-32.7)	0.14	0.44 (0.1-1.3)	0.15
Low vs High Adherence	0.32 (0.0-1.7)	0.22	0.90 (0.1-5.5)	0.91	0.85 (0.0-6.7)	0.88	1.82 (0.0-28.1)	0.66	1.18 (0.2-5.5)	0.82
FM										
Low vs Moderate Adherence	1.76 (0.8-3.8)	0.15	1.09 (0.5-2.1)	0.78	1.17 (0.5-2.5)	0.68	0.89 (0.4-1.9)	0.77	0.57 (0.2-1.1)	0.11
Low vs High Adherence	0.38 (0.0-2.3)	0.38	1.10 (0.2-4.2)	0.88	0.48 (0.0-3.1)	0.51	1.16 (0.2-4.8)	0.83	1.68 (0.3-11.8)	0.53

**model adjusted for age, sex, and educational level*

Abbreviations: Body Mass Index (BMI), Fibromyalgia (FM), Psoriatic Arthritis (PsA), Rheumatoid Arthritis (RA)

Supplementary table S2. Association between PREDIMED score and cardiovascular risk factors/bowel habits

	Smoking habit		BMI > 25 kg/m²		Hypertension		Dyslipidemia		Irregular bowel habit	
PREDIMED score	OR (CI 95%)	*p-value**	OR (CI 95%)	*p-value**	OR (CI 95%)	*p-value**	OR (CI 95%)	*p-value**	OR (CI 95%)	*p-value**
Total population	0.91 (0.8-1.0)	0.13	1.03 (0.9-1.1)	0.45	0.99 (0.8-1.1)	0.92	0.98 (0.8-1.1)	0.87	0.94 (0.8-1.0)	0.29
RA	0.89 (0.7-1.0)	0.28	1.05 (0.8-1.2)	0.54	1.02 (0.8-1.2)	0.75	0.97 (0.7-1.1)	0.77	0.88 (0.7-1.0)	0.15
PsA	0.87 (0.6-1.0)	0.24	1.03 (0.8-1.3)	0.74	1.08 (0.8-1.4)	0.51	1.06 (0.7-1.4)	0.69	1.03 (0.8-1.2)	0.74
FM	0.98 (0.8-1.1)	0.83	1.02 (0.8-1.1)	0.78	0.93 (0.7-1.1)	0.50	1.02 (0.8-1.2)	0.74	0.97 (0.8-1.1)	0.77

**model adjusted for age, sex, and educational level*

Abbreviations: Body Mass Index (BMI), Fibromyalgia (FM), Psoriatic Arthritis (PsA), Rheumatoid Arthritis (RA)

Supplementary table S3. Association between PREDIMED score and disease activity or severity between each diagnostic group

	PREDIMED score	
	β (CI 95%)	*p-value**
**RA**		
DAS28-CRP	-0.06 (-0.16; 0.02)	0.15
PtGA	-0.05 (-0.3; -0.19)	0.65
VAS pain	0.02 (-0.20; 0.24)	0.83
**PsA**		
DAPSA	-0.29 (-1.09; 0.50)	0.45
PtGA	-0.07 (-0.37; 0.23)	0.63
VAS pain	-0.04 (-0.36; 0.27)	0.76
**FM**		
FIQR	**-1.96 (-3.62; -0.30)**	**0.02**
PDS	**-0.64 (-1.12; -0.16)**	**0.009**

***model adjusted for age, sex, BMI, smoking habit and educational level*

Abbreviations: Disease Activity Score28-C Reactive Protein (DAS28-CRP), Disease Activity Index for Psoriatic Arthritis (DAPSA), Fibromyalgia (FM), Revised Fibromyalgia Impact Questionnaire (FIQR), Patient Global Assessment (PtGA), Polysymptomatic Distress Scale (PDS), PREvención con DIeta MEDiterránea (PREDIMED), Psoriatic Arthritis (PsA), Rheumatoid Arthritis (RA), Visual Analogical Scale (VAS)
